# Rituximab combined with intravenous immunoglobulin in autoimmune diseases: a systematic review

**DOI:** 10.1186/s42358-025-00450-x

**Published:** 2025-03-25

**Authors:** Jozélio Freire de Carvalho, Thelma Laroca Skare

**Affiliations:** 1 Núcleo de Pesquisa em Doenças Crônicas não Transmissíveis (NUPEC), School of Nutrition, Federal University of Bahia, R. Basílio da Gama, 200 – Canela, Salvador, Bahia 40110-040 Brazil; 2 Serviço de Reumatologia. Hospital Universitário Evangélico Mackenzie, Curitiba, PR Brazil

**Keywords:** Rituximab, Intravenous immunoglobulin, IVIg, Autoimmune disease, Pemphigus

## Abstract

**Background:**

Although using Rituximab (RTX) and intravenous immunoglobulin (IVIg) alone or sequentially is a well-established treatment for several autoimmune diseases, the combination of these two forms of therapy is still rare, and its use is poorly studied.

**Aim:**

To perform a systematic review on the use of RTX associated with IVIG in autoimmune conditions.

**Methods:**

PubMed/MEDLINE, EMBASE, and Scielo databases were screened for articles on RTX plus IVIg in autoimmune diseases until May 2024.

**Results:**

The review encompassed 21 studies evaluating RTX and IVIg for autoimmune diseases. Ten studies focused on pemphigus, involving 85 patients with diverse subtypes (47 pemphigus vulgaris, 27 pemphigoids, and 11 other variants). Most were case reports or series, with one retrospective study including controls. Positive outcomes were reported across all but one case of paraneoplastic pemphigus. Infections, such as P. jirovecii pneumonia, were noted in three studies, highlighting a potential risk. The other 11 studies involved 24 patients with conditions like polyneuropathies, lupus with CNS involvement, and neuromyelitis optica. While most reported favorable outcomes, one trial on IVIg-dependent polyneuropathies found RTX ineffective in reducing IVIg needs. Adverse events included pneumonia, venous thrombosis with pulmonary embolism, and infusion reactions, demonstrating the need for careful monitoring.

**Conclusion:**

RTX plus IVIg seems to be an alternative option for the treatment of refractory autoimmune diseases. However, more studies with a larger number of participants and in different autoimmune diseases are desired.

**Supplementary Information:**

The online version contains supplementary material available at 10.1186/s42358-025-00450-x.

## Key summary

- Rituximab (RTX) and intravenous immunoglobulin (IVIg) alone or sequentially is a well-established treatment for autoimmune diseases.
- Herein, the authors reviewed the studies that used the combination of RTX plus IVIg in autoimmune conditions.
- Pemphigus, polyneuropathies, lupus, Susac’s syndrome, neuromyelitis optica and acquired factor 8 inhibitor were the studied diseases.
- The outcomes were good and the treatment was well tolerated.

**Supplementary Information:**

The online version contains supplementary material available at 10.1186/s42358-025-00450-x.

## Introduction

Rituximab (RTX) is a monoclonal antibody directed against B lymphocytes, primarily used to treat lymphoma and autoimmune diseases, including rheumatoid arthritis (RA), systemic lupus erythematosus (SLE), scleroderma (SSc), and others. The Fab portion of this antibody identifies a sequence of four amino acids of the CD20, a transmembrane protein, leading to a significant depletion of peripheral B cells, which play a crucial role in immune responses and the production of antibodies [1, 2]. Rituximab has emerged as a promising therapeutic option for ameliorating several autoimmune diseases that are presumed to be B-cell driven [3].

Intravenous immunoglobulin (IVIg) is a pool of immunoglobulins from thousands of blood donors with immunomodulatory properties in autoantibody-mediated, T-cell-mediated autoimmune disorders and systemic inflammatory diseases [4]. It controls T and B lymphocytes’ activation and effector functions, interferes with antigen presentation, neutralizes pathogenic autoantibodies, and has an anti-inflammatory effect due to its interaction with cytokines, complement, and endothelial cells [5].

Both drugs are used separately or sequentially to treat diseases refractory to other treatments or where conventional therapies result in unacceptable side effects [6–8]. The combination of these two drugs (RTX plus IVIg) was first used in highly human leucocyte antigen (HLA)-sensitized patients to reduce anti-HLA antibody levels, allowing kidney transplantation [9], and its use has spread to other forms of refractory autoimmune diseases. Nevertheless, there are few reports on the safety and efficiency of using this combination in autoimmune diseases.

This article aims to perform a systematic review of the data on RTX plus IVIg in autoimmune diseases.

## Methods

This systematic review was conducted in accordance with internationally recognized guidelines for systematic reviews, using the PRISMA (Preferred Reporting Items for Systematic Reviews and Meta-Analyses) methodology [10]. The literature search was performed across three major databases: PubMed, Scielo, and Embase, until May 2024. No language restrictions were applied.

### Search strategy

The following specific MeSH entry terms: “Intravenous immunoglobulin” AND “rituximab” AND “autoimmune” were used. These terms were selected to maximize sensitivity and specificity, ensuring the inclusion of relevant studies.

**Inclusion criteria**: Studies involving adult patients (age ≥ 18 years); and prospective studies investigating RTX associated with IVIg as treatment of autoimmune diseases.

**Exclusion criteria**: Narrative or systematic reviews, protocols, opinion papers, editorials; and preclinical studies, including in vivo and in vitro research.

### Study selection and data extraction

Articles identified during the search were initially screened by title and abstract to determine eligibility. Full-text articles of potentially relevant studies were subsequently reviewed. Two reviewers (JFC and TLS) conducted the screening and data extraction to minimize bias. If there were any discordance, a third reviewer would be included.

### Extracted data included

Sample size and demographic characteristics of participants; diagnosis of the autoimmune disease; dosage and duration of RTX and IVIg therapy; methods used to assess outcomes, such as disease activity indices, and clinical parameters; and reported adverse events.

## Results

**Fig. 1:**
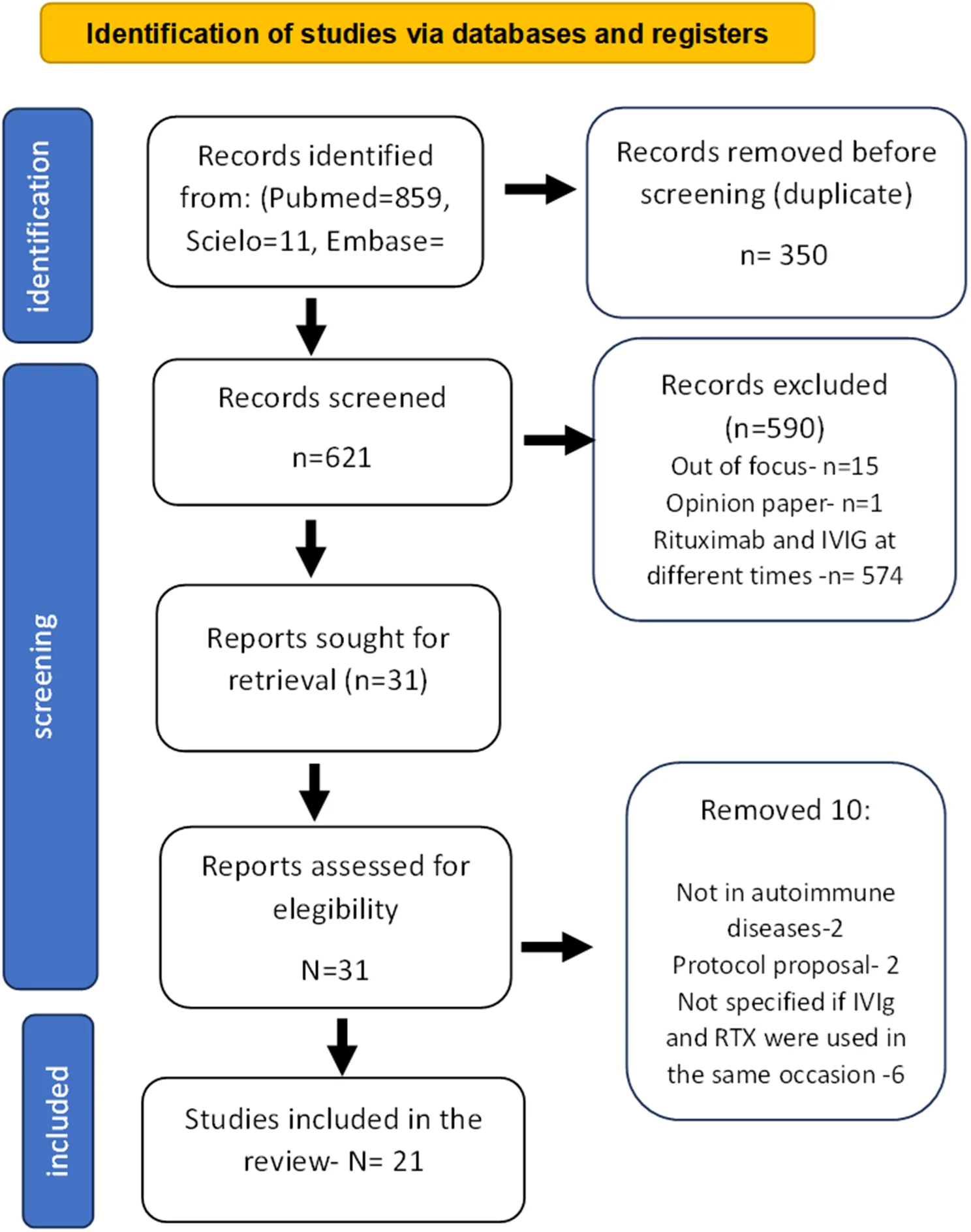
Flowchart of the included studies

Figure 1 shows the flowchart of the included studies.

Twenty-one papers were selected for review [11–31].

Table 1 describes the 10 articles on RTX and IVIg used for pemphigus treatment [11–20]. The countries that reported those articles were the United States (*n* = 5) [11, 12, 14, 17, 19], Germany (*n* = 1) [13], Japan (*n* = 1) [15], UK (*n* = 1) [18], India (*n* = 1) [20], Canada (*n* = 1) [31], and Saudi Arabia (*n* = 1) [16], encompassing 85 patients (47 on pemphigus vulgaris; 27 on pemphigoids and 11 with miscellaneous forms of pemphigus). These works were prospective (*n* = 1) [12], retrospective (*n* = 4) [11, 14, 16, 17], and reports of cases or series of cases (*n* = 5) [13, 15, 18–20].

**Table 1:** Studies on rituximab associated with intravenous Immunoglobulin in pemphigus

Author, year	Study design	Local	*N*	Age (years old)/ gender	Indication	Disease duration	RTX and IVIg dose	Follow-up	Outcome	Side effects
Ahmed et al., 2006 [12].	Open prospective uncontrolled	USA	11	Mean = 38 yo 55% females	PV	30 to > 96 mo	*Months 1 and 2-* RTX (375 mg/m^2^ once a week for 3 weeks) + IVIg (2 g/kg in the fourth week). *Months 3*,* 4*,* 5*,* and 6* – a single infusion of RTX + 2 g of IVG / month. If the patient was disease-free: − 7 additional IVIg infusions.	37 mo.	All patients had improvement between 3rd and 6th infusions of RTX; Complete clearance between 7th and 9th infusions; 9/11 (82%) had sustained remission (mean of 31.1 mo). All immunosuppressive drugs were discontinued.	None
Mühlhoff and Megahed, 2007 [13].	Case report	Germany	1	44	PV	4 years	RTX- 375 mg/m^2^ once a week for 3 weeks. In the fourth week, IVIg 500 mg/kg/day for 3 days and then RTX plus IVIg per month for 9 months	ND	Clinical improvement and reduction in prednisolone to 5 mg/day. Significant decrease in anti-desmoglein 1 and 3 titers.	None
Foster et al., 2010 [11].	Retrospective, Comparative Interventional case series	USA	12 - divided into 2 groups	*Group 1*- mean age = 57 yo; *Group 2*- mean age = 63 yo 50% females	Ocular cicatricial pemphigoid	*Group 1*→ 21 mo; *Group 2*→18.5 mo.	*Group1*→6 patients received RTX (375 mg/m^2^/week for 8 weeks followed by once monthly for 4 mo) and IVIg (2 g/kg per cycle →1 cycle/month until B cell level returned to normal and after that at intervals of 6,8,10,12,14 and 16 weeks) *Group 2* →6 patients received aggressive immunosuppression.	*Group 1*→ median 55.5 mo *Group 2*→ median 27.5 mo.	*Group 1*- no further deterioration of visual acuity; *Group 2*- decrease in visual acuity; all patients were blind at the end of the study	None
Feldman et al., 2011 [14].	Retrospective	USA	19	Mean = 51 ± 9 yo 97% females	PV	ND	RTX- 375 mg/m^2^ once a week for 8 weeks and then every 4 weeks until week 24 weeks; IVIg 2 g/kg once a month.	29.6 mo in the LTR group; 40 mo in the relapse group.	11/19 (58%) achieved long-term clinical remission; 8/19 had 15 relapses.	ND
Ahmed et al., 2015 [17].	Retrospective	USA	12	Mean age – 68.2 yo; 58.3% females.	Bullous pemphigoid	36.5 mo	*Phase 1*- IVIg 2 g/kg/cycle - at monthly intervals + 8 infusions of RTX (375mg/m^2^) weekly; *Phase 2*- IVIG multiple cycles at monthly intervals depending on CD19 + B cell count; *Phase 3*–6 cycles of IVIg given at 6-, 8-, 10-, 12-, and 14-week intervals	Mean = 73.8 mo	Control of disease activity was observed in a mean of 4.15 (range 2.3–6.8) weeks; Two patients relapsed (one after 22 mo and the other after 18 mo) but refused additional infusion of RTX.	None
Namba et al., 2016 [15].	Case report	Japan	1	59 Female	Paraneoplastic pemphigus + bronchiolitis obliterans + B cell lymphoma	1 mo	375 mg/m^2^ weekly for 3 weeks for 5 cycles; IVIg 400 mg/kg over 5 days once a month for 3 cycles, associated with steroid (pulse) and cyclosporin + R-CHOP for lymphoma	6 mo	No clinical response (skin but not mucosal improvement); Patient died after six months from bronchiolitis obliterans	Infections (bacterial, fungal, and cytomegalovirus).
Steger et al., 2016 [18].	Case series	UK	4	*Pat1*: 54 yo; male; *Pat 2*: 83 yo; male; *Pat 3*: 63 yo, female; *Pat 4*: 21 yo, male	Autoimmune cicatricial conjunctivitis (3 with mucous membrane pemphigoid and 1 with linear IgA disease)	*Pat 1*: 17 mo; *Pat 2*: 3 mo; *Pat 3*: 0; *Pat 4*: 2 mo	RTX: 1.000 mg- twice with 2 weeks interval; IVIg: 2 g/kg divided into 3 daily doses. *Pat1*: 2 courses of RTX + 3 courses of IVIg with 1 st infusion of RTX and 6 courses parallel to 2nd infusion of RTX. *Pat 2 and 4*: 1 course of RTX + 2 courses of IVIg.	36, 32, 65, and 42 mo, respectively.	Conjunctival inflammation settled in all cases; fornical shortening continued throughout 8–12 mo without the active conjunctival disease. Reasonable visual acuities were maintained in all but 1 patient.	*Pat 2*- developed pneumonia + life-threatening septicemia. Same patient- corneal infection with corneal melting.
Hamadah et al., 2018 [16].	Retrospective	Saudi Arabia	23 (*)	Mean = 45 yo; 48% females	15 PV, 5 PF, 1 each with: -P. erythematosus, -P. vegetans, -P. herpetiformis.	31 mo	RTX- 375 mg/m^2^ once a week for 3 weeks. In the fourth week, IVIg 2 g/kg over 3 days	69 (8-126) months	90.5% achieved complete remission	17%- infusion reactions. 8% -cellulitis, 8%-cytopenias, 4% - molluscum contagiosum; 1 patient died from MI (not related to therapy).
Narayanan et al., 2021 [20].	Case report	India	1	32 yo Female	Pemphigoid gestationis	From 2 mo before delivery to 6 mo post-partum	IVIg- 30 g/day for 5 days (3 courses 2 weeks apart); RTX – 1 g, 2 doses, 2 weeks apart) Azathioprine after second course of IVIg	6 mo	All the lesions had resolved one month after the 3rd course of IVIg.	ND
Foschi et al., 2022 [19].	Case report	USA	1	73 yo Male	PV	10 years	375 mg/m^2^ RTX/week for 3 weeks. After the 4th week, IVIG (2 g/kg/month) + methylprednisolone (96 mg)	ND	PV improved, and glucocorticoid was tapered.	Deep vein thrombosis; Pneumonia by P. carinii

yo=- years old; mo = months; P = pemphigus; PF: pemphigus foliaceus, PV: pemphigus vulgaris, PNP: paraneoplastic pemphigus; RTX- rituximab; IVIg = intravenous immunoglobulin; LTR: long-term remission

(*) only 6 patients (4 with PV and 2 with PF) started treatment with combination therapy; 9.1% of the total sample required combination therapy to treat relapses. MI = myocardial infarction

All studies had a small number of patients; the biggest had *n* = 23 [16]. Only one retrospective study had a comparison with controls [11]. Also, but one study found positive clinical results of this form of treatment with improvement in skin pemphigus lesions; the negative clinical response were from a case report on paraneoplastic pemphigus [15].

The combination was well tolerated, but infections were a problem in 3 studies, including one with pneumonia by P. jiroveci [14, 16, 19].

Table 2 summarizes the 11 studies in other autoimmune entities encompassing another 24 patients [21–31]. They were from the USA (*n* = 5) [21, 23, 24, 27, 28], Spain (*n* = 2) [22, 29], Australia (*n* = 1) [25], Turkey (*n* = 1) [26], Saudi Arabia (*n* = 1) [30], and Canada (*n* = 1) [31]. There was 1 small prospective open-label trial [23] with 6 individuals with IVIG dependent polyneuropathies; all others were case or series of case reports [21, 22, 24–31]. They studied polyneuropathies (*n* = 3) [23, 27, 28], Lupus with central nervous system manifestations (*n* = 2) [25, 30], Susac syndrome (*n* = 1) [24], neuromyelitis optica (*n* = 1) [22], acquired factor 8 inhibitor (*n* = 1) [21], thrombotic thrombocytopenic purpura (*n* = 1) [29], autoimmune hemolytic anemia (*n* = 1) [31], and bullous epidermolysis acquisita (*n* = 1) [26]. The results were clinically positive in all but one observation: the open trial with IVIG-dependent polyneuropathies in which the addition of rituximab did not reduce IVIg requirements in most patients [23]. Patients with hematological manifestations had clinical and blood cell count improvements. Most neurological studies showed a clinical improvement and a few article showed MRI improvement and autoantibodies decrease (e.g. anti-aquaporine).

**Table 2:** Studies of rituximab associated with intravenous Immunoglobulin in several autoimmune conditions

Author, reference	Study design	Country	*N*	Age (years old)/gender	Disease	Disease duration	RTX dose (mg/day), IVIg dose	Follow-up	Outcome	Side effects
Muzaffar et al., 2012 [21].	Case report	USA	1	65 yo Male	Acquired factor VIII inhibitor	3 days	IVIg (8 doses)-1 g/kg/day) RTX − 750 mg /m^2^ – 4 weekly doses.	4 mo	Patients went into remission with disappearance of acquired factor VIII inhibitor	ND
Andres et al., 2014 [22].	Case report	Spain	1	17 yo; Female	Neuromyelitis optica	5 years	RTX-375 mg/m^2^ for 2 doses; IVIg- 400 mg/ kg/day for 5 days	2 years	After 4 months, the neurological examination was normal; improvements in MRI and reduction in anti-aquaporin-4 titers.	None
Gorson et al., 2015 [23].	Prospective open-label	USA	6	Mean age – 68.2 yo; ND	IVIg-dependent autoimmune polyneuropathy	ND	RTX (375 mg/ m2 /week) for 4 weeks + baseline IVIg.	*1 year*	RTX did not reduce IVIg requirements in the majority of patients with IVIg-dependent, immune-mediated polyneuropathies.	None
Gertner et al., 2016 [24].	Case report	USA	1	22 yo; male	Susac syndrome	2 weeks	IVIg- 500 mg/kg – day 1,3 and 4; RTX- 1000 mg – day 2 Maintenance with IVIg, AAS, and azathioprine.	6 mo	After the first month of treatment, he was able to resume all his daily activities without difficulty or recurrence	ND
Watson et al., 2017 [25].	Case report	Australia	1	64 yo Male	Neuropsychiatric systemic Lupus erythematosus	ND	ND	ND	The neuropsychiatric syndrome settled, and the disease activity reduced.	ND.
Oktem et al., 2017 [26].	Case series	Turkey	5	Mean = 59 yo; 2 females; 3 males.	Epidermolysis bullosa acquisita	Mean = 8.8 y.	*Pat. 1*,*2*,*3 and 5* - RTX (375mg/m^2^) 4 weekly cycles + IVIG (2 g/kg/ month) monthly (24, 25, 26, and 10 cycles, respectively); *Pat.4* – RTX- 2 cycles of 3 consecutive weeks + IVIG monthly- (12 cycles)	Mean = 22.6 mo.	Improvement of skin and mucosal lesions.	Fever, shivering, and urticaria-like eruption in pat 0.2.
Birnbaum et al., 2017 [27].	Case report	USA	1	35 yo; female	Sjögren with sensory neuronopathy	2 mo	RTX − 375mg/m^2^ − 2 doses weekly followed by 1 dose RTX every 10 weeks; + IVIg (0.5 g/kg) every 2–3 weeks. -	23 mo	After 6 RTX doses- the patient, who used a wheelchair, became able to ambulate with a cane.	ND
Lima et al., 2022 [28].	Case series	USA	3	32 yo, 40 yo,61 yo 100% males	Refractory vasculitis neuropathy (1 granulomatosis with poliangeiitis)	*Pat1*-2 y; *Pat 2*–6 y; *Pat 3*–4 y;	*Pat 1*: RTX 1 g, twice (2 weeks apart) + 1 g every 4 months; IVIg 400 mg/kg monthly; MMF 2 g/day. *Pat 2*: RTX- ND; IVIg- 1 g/kg/every 4 weeks; *Pat 3*: RTX- ND; IVIg 2 g/kg over 5 days (used twice)	*Pat 1-* 6mo; *Pat 2*–3 y; *Pat 3* - ND	*Pat 1*- Regained ability to ambulate with a walker and recovery sensation to pinprick/light touch to 3 cm below the elbow and just above the knees. *Pat 2* - Improvement; the patient was able to resume exercises; *Pat 3*-Improvement of paresthesias.	*Pat 1 and 2-* none *Pat 3* – pneumonia; venous thrombosis + pulmonary embolism
Cid et al., 2021 [29].	Case series	Spain	3	48 yo (female), 61 yo (female); 24 yo (male)	Autoimmune thrombotic thrombocytopenic purpura	1, 1, and 7 days,	RTX- 375 mg/m2/week for 4 weeks IVIg 100–400 mg/Kg Associated with plasma exchange, steroids, and caplacizumab	6 months, 3 months, and 3 months, respectively.	*Pat. 1*: improvement with normal platelet count, ADAMTS 13 activity 100%, and negative anti-ADAMTS 13. *Pat. 2*: improvement with normal platelet count, ADAMTS 13 activity 19%, and negative anti-ADAMTS 13. *Pat. 3*: improvement with normal platelet count, ADAMTS 13 activity 100%, and negative anti-ADAMTS 13.	None
Cheikh et al., 2022 [30].	Case report	Saudi Arabia	1	33 yo; male	Neuropsychiatric Lupus + Lupus nephritis CNS symptoms- one week after extubating from a septic shock	1 year	IVIg – 0,4 g/kg/day for 5 days + RTX- 500 mg once + methylprednisolone 1 g/5 days	4 mo	Improvement of CNS symptoms after 5 days. 3 weeks after IVIg and RTX, he was started on MMF and antimalarials. 4 mo later - marked improvement of CNS manifestations and remission of nephritis.	ND
Castillo et al., 2022 [31].	Case report	Canada	1	23 yo; Male	Autoimmune hemolytic anemia with Epstein Barr proliferation in a post-heart transplant	1 week	IVIg- 1 g/kg for 2 days; RTX (one dose)- 700 mg	5 mo	Improvement; the hemoglobin went from 3.1 g/dL to 14.8 g/dL	MSSA pneumonia

Pat = patient; n = number; yo = years old; y = years; mo = months; Pat = patient; IVIg = intravenous immunoglobulin; RTX = rituximab; MSSA = methicillin susceptible Staphylococcus aureus

The treatment was well tolerated despite 2 cases of pneumonia [28, 31], one of venous thrombosis complicated with pulmonary embolism [28], and one with infusion reactions [26].

## Discussion

The evidence about the efficiency of combining RTX with IVIg is low and supported mainly by case descriptions. However, the preliminary results suggest that this field deserves further investigation, especially because this combination is used in severely ill patients in whom other drugs have been ineffective.

Autoimmune diseases associated with autoantibodies are presumed to be B cell-determined. Rituximab is a B cell depletion therapy that targets the CD20 molecule expressed on the surface of B lymphocytes from the pre-B cell stage to memory B lymphocytes, sparing stem cells and plasma cells. By depleting autoreactive B cells, it reduces the production of pathogenic autoantibodies [32].

Intravenous immunoglobulin (IVIg) exerts several anti-inflammatory and immunomodulatory effects, but not all mechanisms have been fully elucidated. It interacts with complement fragments C3b and C4b in the complement cascade, preventing the formation of the membrane attack complex and avoiding complement-mediated cell death and tissue damage [33–35]. It also counteracts C3a and C5a anaphylatoxins via an F(ab′)2-mediated mechanism. Furthermore, IVIg inhibits autoantibodies through idiotype networks [36]. Additionally, IVIg can bind to the neonatal Fc receptor (FcRn), blocking this protective receptor, which normally prevents the catabolism of IgG, thereby inducing the clearance of antibodies, including pathogenic ones [37, 38]. It also upregulates the inhibitory receptor FcγRIIB, which increases the threshold for immune-complex-mediated activation of innate immune cells [39].

Moreover, IVIg exerts inhibitory actions on monocytes and macrophages, inducing the release of anti-inflammatory cytokines such as IL-1 receptor antagonist (IL-1RA), TGF-β, and IL-10 [40, 41]. It also impedes the proliferation and antigen-presenting activities of B cells, as well as their activation through IL-4, CD40, TLR, and BCR signaling [42, 43]. However, while these immunomodulatory actions are well-documented, the complete range of IVIg’s mechanisms of action, particularly in autoimmune contexts, remains an area of ongoing research.

Rituximab treatment brings an essential risk for infection. Studies done on RA patients described that 10% of patients using RTX have hypogammaglobulinemia after the first course and 30% after the fourth course [44]. IVIg therapy prevents and fights severe infections [45, 46]. So, this combination is not only synergistic in treating autoimmune diseases, but IVIG helps prevent the collateral effects of RTX, becoming an ideal partner in this context.

The cases described are mainly on the several forms of pemphigus, which are severe diseases with a high risk of infection due to mucosal and skin blistering. In the 85 patients with pemphigus, few infections were seen with the use of this combination: 2 cases of pneumonia [18, 19], 1 ocular infection [18], 1 molluscum contagiosum [16], and 2 skin infection [16]. Also, several bacterial and fungal infections were seen in one patient with paraneoplastic pemphigus, who also received chemotherapy for lymphoma [15]. Two deaths were observed in this group, but both were unrelated to the treatment [15, 16]. In the group of other autoimmune diseases, two cases of pneumonia were seen among the 24 studied patients, and both were resolved with antibiotic treatment [28, 31].

The small study by Foster et al. [11] was particularly striking because it showed that this combination of drugs can prevent scarring blindness secondary to pemphigus mucosal involvement, while conventional immunosuppression cannot.

Some protocols for RTX and IVIg combination have been proposed [46, 47], and they may uniformize the doses and timing used, allowing better analysis of results in the future.

A limitation of this analysis is that most included studies are case reports, case series, or retrospective analyses, generating a limited number of patients. This type of evidence is, therefore, generally lower in quality, and the present findings should be interpreted with caution.

In conclusion, this review of RTX/IVIg shows that this therapy seems to effectively treat refractory autoimmune disorders with mild/moderate adverse effects. Future studies using RTX/IVIg using a significant number of autoimmune conditions in large cohorts of patients with autoimmune diseases are desired.

## Electronic supplementary material

Below is the link to the electronic supplementary material.

Supplementary Material 1

Supplementary Material 2

## Data Availability

No datasets were generated or analysed during the current study.

## Declarations

### Medical writing

Both authors wrote all this manuscript.
